# Gatherer ancestry associated with national happiness

**DOI:** 10.1371/journal.pone.0336161

**Published:** 2026-01-21

**Authors:** Matthew Frederick Basilico

**Affiliations:** 1 Department of Economics, Harvard University Faculty of Arts and Sciences, Cambridge, Massachusetts, United States of America; 2 Department of Global Health and Social Medicine, Harvard Medical School, Boston, Massachusetts, United States of America; 3 Mass General Brigham McLean Hospital, Belmont, Massachusetts, United States of America; 4 Department of Psychiatry, Harvard Medical School, Boston, Massachusetts, United States of America; Tel Aviv university, ISRAEL

## Abstract

Efforts to improve human emotional wellbeing through economic growth have seen varied success. One interpretation of the lack of wellbeing returns to economic growth is that humans may have been more emotionally suited to patterns of life in pre-agricultural societies. This study examines the hypothesis, dating to Rousseau, that descendants of hunter-gatherer societies have higher levels of subjective wellbeing. It utilizes data from 1265 small scale societies in the Murdock Ethnographic Atlas to construct a country-level measure of gatherer ancestry. Average country-level happiness and life satisfaction were derived from the World Values Survey which covered 104 countries from 1981–2014. Gatherer ancestry was significantly associated with happiness, controlling for contemporary income per capita (beta = 13.58; standard error = 3.0, R^2^ = 11.8%, p < 0.01). Results were robust to an extensive list of historical and contemporary controls. The findings are consistent with the hypothesis that gatherer lifestyle organization may hold insights for human emotional wellbeing.

## Introduction

The relationship between human economic organization and societal emotional wellbeing continues to hold many puzzles. The well-known Easterlin Paradox is the documentation of a pattern that country-level economic growth and happiness are not meaningfully correlated in the long-run since data became available in the 1960s, a relationship that is of increasing concern given the consequences of economic growth for the environment [1,2]. Income and emotional wellbeing are also tenuously related for middle to high income individuals in the United States and Europe [3,4]. Emotional distress among young people has appeared at new levels in the United States despite historically unprecedented average levels of consumption and effective material living standards [5,6]. The notion that individual income improvements should lead to wellbeing improvements remains central to economic modelling and policymaking [7,8].

Other prominent social theorists, such as Jean-Jacques Rousseau, have hypothesized that hunter-gatherer social organization may hold benefits for human emotional wellbeing despite income limitations. Rousseau hypothesized that these benefits are eroded in societies with a higher degree of division of labor and thus inequality [9]. Contemporary evolutionary psychologists, such as Randolph Nesse, Glenn Geher and Nikhil Chaudhary, have similarly hypothesized that high prevalence rates of emotional distress, anxiety and depression may be the result of a “mismatch” between human emotional systems which evolved in the environment of small-scale societies and contemporary human environments (e.g., typically hierarchical, non-subsistence, including agricultural and industrial organization) [10–15]. The work of these theorists leads to the hypothesis that wellbeing levels may be higher on average in small-scale hunter-gatherer societies than in contemporary industrialized societies, despite the higher consumption levels of the latter. The hypothesis has been given empirical support by several recent studies. For example, Miñarro and colleagues (2021) find higher levels of subjective wellbeing in a sample of minimally-monetized societies compared to high income countries [16]. Reyes-Garcia and colleagues (2021) find high levels of subjective wellbeing in a sample of 474 adults from 3 small-scale societies (Baka, Punan and Tsimane’), while Galbraith and colleagues (2024) similarly find high levels of life satisfaction in a sample of 19 small-scale societies [16,17]. Fedurek and colleagues (2023) find that status does not correlate with physiologic stress levels in a sample of Hadza hunter-gatherer men, in contrast to consistent findings in high-income societies of a correlation between stress and status [18]. Gurven and colleagues (2025) examine patterns of subjective wellbeing across the lifecycle in three small-scale societies, discovering departures from the typical age-patterns observed in high-income country samples [19]. The fields of Positive Evolutionary Psychology and Evolutionary Psychiatry have expanded rapidly over the last decade around the insight that human affect may be naturally attuned to particular historical small-scale societies [10,12,20–24].

The present study seeks to enhance our understanding of how wellbeing levels in small-scale societies may relate to contemporary, country-level subjective wellbeing. It investigates the hypothesis that societies with a higher levels of ancestry from hunter-gatherer societies may be associated different rates of subjective wellbeing. Recent innovations in historical economics have allowed scholars to link features of ancestral societies to modern-day populations, and investigate relationships between historical economic organization and modern-day beliefs [25–27]. One of the initial works in this space examined how ancestral cultures that used plough-based agriculture had a higher gender-based segregation of labor, and modern-day populations with a higher fraction of descendants from plough-based societies have more unequal gender norms today [28]. Since this paper, over a dozen works in historical economics have uncovered mechanisms of cultural persistence, linking features of pre-industrial societies to contemporary economic and cultural outcomes [26,27]. While it has been well-utilized in several influential economics papers, the approach does have important limitations, such as assigning country-level cultural ancestry based on language group, reducing important sources of variation [29,30]. Nevertheless, the approach makes possible a degree of mapping from the features of ancestral societies to help characterize the influence on contemporary populations.

The Ethnographic Atlas tables, compiled in 1967 by Anthropologist George Peter Murdock, “incorporate nearly 50,000 distinct items of information” based upon earliest available ethnographic sources [31]. The data in the Ethnographic Atlas reflects understanding of human social organization for several generations up to the 20^th^ century. Although most humans before the neolithic revolution (circa 10,000 BCE) are believed to have lived in small-scale societies, Henrich and colleagues have noted that the substantial heterogeneity of human societal organization over the past several millennia, including pastoral, agricultural and industrial practices, has led to important variation in cultural norms [32–34]. Hence, we are able to ask the question: is a higher fraction of recent ancestral descendants from hunter-gatherer societies associated with contemporary average wellbeing, controlling for income? As noted above, several investigations have directly sampled wellbeing levels in hunter- or gatherer-based contemporary societies and typically found higher levels of subjective wellbeing than industrialized country samples, although these studies are naturally limited by the remaining prevalence of this form of social organization [35–37]. The accumulating body of evidence in historical economics suggests, if they existed, some of these wellbeing advantages could transmit to modern day populations through channels of cultural persistence [38,39].

The present study investigates this question using contemporary tools of historical economics, including linking the Murdock Ethnographic Atlas to countries, and utilizing cross-country subjective wellbeing data available from the World Values Survey and economic controls available from the World Bank Group’s World Development Indicators.

## Methods

This study examines how the variance in measured average population-level happiness varies with the fraction of ancestry from hunter-predominant, gather-predominant, or agricultural-predominant societies. We follow the methodology utilized in Alesina, Giuilano 2013 which linked the Murdock Ethnographic Atlas data to country-level populations using the steps outlined below [28].

The *Ethnographic Atlas* was constructed in 1967 by George Peter Murdock, coding data on 1265 small scale societies utilizing earliest-available ethnographic evidence. The dataset includes over 100 variables, including coding economic organization features such as predominant mode of food production, levels of political hierarchy, and kinship practices [40]. Within the ethnographic atlas, variable *v42* indicates the predominant mode of food production in society. Here, “Gathering contributes most” (103 of 1265 ethnic groups) is coded as Gathering Predominant, “Hunting contributes most” is coded as Hunting Predominant (75 of 1265 ethnic groups). “Fishing contributes most” is coded as Fishing Predominant (114 of 1265 ethnic groups). For comparison, the majority of societies are coded as “Intensive agriculture contributes most” (270 groups), “Extensive agriculture contributes most” (475 groups), “Agriculture contributes most, type unknown” (86 groups), or “Pastoralism contributes most” (77 groups). Remaining observations are either more than one equal sources (64 groups), or missing observation (1 group). The ethnographic atlas data is then linked to the *Ethnologue: Languages of the World* dataset, which maps the prevalence of 7612 languages globally. Each language in the *Ethnologue* is linked to an observed society in the Ethnographic atlas. Finally, the *Ethnologue* is linked to modern nation states by the percentage of the population speaking the language coded*.* Following Giuilano and Nunn 2018, each variable is then rescaled to adjust for missing values. Namely, *v42* group 1 denotes missing values (1 of 1265 ethnic groups). The resulting country-level estimate of “Gathering Predominant Ancestry” is thus the fraction of contemporary ancestry estimated to have descended from Gathering predominant societies (*v42*). Country-level “Hunting Predominant Ancestry” is estimated in a similar fashion, and the two fractions are summed to create the variable “Hunting or Gathering Predominant Ancestry.” This technique was pioneered by Alesina, Giuiliano and Nunn 2013, and a mapping to country-level data is available from Giuiliano and Nunn 2018 [26,28]. The result of this mapping is an estimate of the country-level fraction of ancestral dependence on gathering or hunting. A similar procedure is used to create the ancestral fractions of control variables used from the ethnographic atlas.

Outcome data on happiness and life satisfaction at the country level comes from the World Values Survey (WVS) Wave 1–6 Key Aggregates, available online through worldvaluessurvey.org. Waves 1–6 covered 108 countries at least once from 1981–2014. Happiness was recorded in each survey using a four-point scale, according to the question, “taking all things together, would you say you are: very happy, rather happy, not very happy, not at all happy.” Life satisfaction is recorded in each survey using a scale from 0 to 10, indicating the degree to which respondents “are satisfied with their life as a whole.” Answers are rescaled from 0 to 1.0, utilizing fractions for intermediate responses [41]. Average happiness and life satisfaction are reported at the country-level for the most recent wave available in the dataset.

To estimate the relationship between the fraction of ancestral population from hunter and gatherer populations and subjective wellbeing, ordinary least squares (OLS) regression with heteroskedasticity robust standard errors was conducted using Stata 18 (StataCorp 2024) [42]. Our baseline specification includes contemporary and historical controls as well as continent fixed effects. Contemporary controls include GDP per capita, using 2019 estimates available publicly from the World Bank Group’s World Development Indicators [43]. Historical controls include observables for key dimensions of societal variation available in the Murdock Atlas: levels of political hierarchy, presence of polygyny, patrilineal descent, and kinship score. Kinship score is an aggregate scale created from four Murdock Atlas variables reflecting degree of kinship tightness, including nuclear family, post-wedding residence, unilineal descent, segmented clans. The scale was developed by economist Benjamin Enke is publicly available from his 2019 paper Kinship, Cooperation and the Evolution of Moral Systems [30]. The linkage of these ancestral characteristics to the contemporary country-level was accomplished through the same procedure for the ancestral gathering variable outlined above. The practice of using a baseline specification with contemporary GDP per capita and selected historical controls was established by Alesina et al 2013 and has become standard practice across over 20 papers in the historical economics literature [27,28]. Coefficient estimates with and without the presence of these controls are presented in the results.

## Results

Ethnographic Atlas and World Values Survey outcome data were matched on n = 102 countries. World Bank data for contemporary controls was matched on n = 100 of the full sample, and a full set of Ethnographic controls, including the Enke Kinship Score, was matched for n = 97 countries. For n = 102 countries with World Values Survey and Ethnographic Atlas observables, average ancestral gatherer fraction was 0.048% (range: 0.0 to 1.26%, std. dev. 0.18%), average hunter ancestry was 0.044% (range: 0.0 to 2.1%, std. dev. 0.24%), average hunter or gatherer ancestry was 0.092% (range: 0.0 to 2.3%, std. dev. 0.31%).

In our baseline specification, the fraction of the population with ancestry from gathering-predominant societies is positively associated with country-level happiness, controlling for contemporary income per capita (beta = 13.58; Standard Error (SE) = 3.0, R^2^ = 11.8%, p < 0.01; Table 1, Column 2). A one standard deviation increase (0.4%) in the fraction of gathering predominant accounts for a 0.05 point increase in average country-level happiness, equivalent to roughly 55% of the standard deviation (0.091 points) of average happiness rates between countries. The fraction of population ancestry from gatherer-predominant populations is positively associated with country-level happiness with or without including controls for GDP per capita, historical controls, or continent fixed effects (Table 2; Columns 2–5).

**Table 1:** Gathering Predominant Ancestry Fraction and Country-Level Happiness.

	(1)	(2)	(3)	(4)	(5)
	Happiness				
Gathering Predominant Ancestry	13.86***	13.58***	10.61***	13.16***	11.53***
	(2.503)	(2.997)	(1.883)	(3.635)	(2.534)
Log GDP Per Capita 2019 (WDI)		0.00751**	0.0136***	0.0143***	0.0184***
		(0.00314)	(0.00411)	(0.00437)	(0.00417)
Average Level of Political Hierarchy				0.0232	0.0250*
				(0.0143)	(0.0131)
Average Settlement Complexity				0.00244	0.00489
				(0.00704)	(0.00808)
Patrilineal Descent				0.0224	0.0239
				(0.0340)	(0.0277)
Matrilineal Descent				−0.125	−0.0803
				(0.0778)	(0.0861)
Polygynous				0.00873	0.0657
				(0.0482)	(0.0660)
Plough Use				−0.170***	−0.168***
				(0.0324)	(0.0340)
Kinship Score				−0.0452	−0.0446
				(0.0484)	(0.0398)
Constant	0.697***	0.558***	0.470***	0.502***	0.373***
	(0.00910)	(0.0624)	(0.0668)	(0.125)	(0.0994)
Observations	102	100	100	97	97
R-squared	0.081	0.118	0.247	0.361	0.488
Continent Fixed Effects			Yes		Yes

Table 1. Results from OLS regression of WVS average country-level Happiness on Gathering Predominant Ancestry (Column 1) as well as including contemporary and historical controls (Columns 2-5). Robust standard errors in parentheses. *** p < 0.01, ** p < 0.05, * p < 0.1.

**Table 2:** Hunting Predominant Ancestry Fraction and Country-Level Happiness.

	(1)	(2)	(3)	(4)	(5)
	Happiness				
Hunting Predominant Ancestry	4.138**	3.754***	−1.028	2.767*	−1.716
	(1.685)	(1.202)	(1.692)	(1.498)	(1.685)
Log GDP Per Capita 2019 (WDI)		0.00737**	0.0137***	0.0138***	0.0178***
		(0.00310)	(0.00410)	(0.00440)	(0.00410)
Average Level of Political Hierarchy				0.0147	0.0230
				(0.0161)	(0.0145)
Average Settlement Complexity				0.00361	0.00596
				(0.00715)	(0.00817)
Patrilineal Descent				0.00812	0.0128
				(0.0347)	(0.0280)
Matrilineal Descent				−0.147*	−0.0909
				(0.0823)	(0.0908)
Polygynous				0.0209	0.0836
				(0.0446)	(0.0636)
Plough Use				−0.160***	−0.159***
				(0.0338)	(0.0335)
Kinship Score				−0.0348	−0.0494
				(0.0497)	(0.0400)
Constant	0.702***	0.564***	0.472***	0.523***	0.388***
	(0.00918)	(0.0618)	(0.0666)	(0.127)	(0.129)
Observations	102	100	100	97	97
R-squared	0.013	0.059	0.211	0.304	0.449
Continent Fixed Effects			Yes		Yes

Table 2. Results from OLS regression of WVS average country-level Happiness on Hunting Predominant Ancestry (Column 1) as well as including contemporary and historical controls (Columns 2-5). Robust standard errors in parentheses. *** p < 0.01, ** p < 0.05, * p < 0.1.

The fraction of population with ancestry from hunter-predominant societies is positively associated with country-level happiness, controlling for contemporary income per capita (beta = 3.75, SE = 1.2, R^2^ = 5.9%, p < 0.01; Table 2, Column 2). The coefficient is approximately 27% as large as the estimated coefficient on gathering-predominant ancestry. However, when including controls for continent fixed effects and historical controls, the results are mostly insignificant (Table 2, Columns 3–5).

The fraction of ancestry from either hunting or gathering-predominant societies was positively associated with country-level happiness, controlling for contemporary income per capita (beta = 6.62, SE = 2.5, R^2^ = 10.1%, p < 0.01; Table 3, Column 2). Results were robust to inclusion of historical controls (Table 3, Column 2) and were no longer significant when including continent fixed effects (Table 3, Columns 3, 5).

**Table 3:** Hunting or Gathering Predominant Ancestry Fraction and Country-Level Happiness.

	(1)	(2)	(3)	(4)	(5)
	Happiness				
Hunting or Gathering Predominant Ancestry	7.225***	6.618***	3.056	5.882**	2.790
	(2.671)	(2.469)	(2.307)	(2.735)	(2.788)
Log GDP Per Capita 2019 (WDI)		0.00721**	0.0134***	0.0144***	0.0178***
		(0.00311)	(0.00410)	(0.00437)	(0.00409)
Average Level of Political Hierarchy				0.0158	0.0211
				(0.0148)	(0.0140)
Average Settlement Complexity				0.00262	0.00549
				(0.00708)	(0.00815)
Patrilineal Descent				0.0122	0.0126
				(0.0338)	(0.0273)
Matrilineal Descent				−0.135	−0.0927
				(0.0805)	(0.0883)
Polygynous				0.0159	0.0778
				(0.0450)	(0.0642)
Plough Use				−0.159***	−0.159***
				(0.0326)	(0.0333)
Kinship Score				−0.0275	−0.0410
				(0.0489)	(0.0401)
Constant	0.697***	0.563***	0.475***	0.506***	0.390***
	(0.00928)	(0.0619)	(0.0665)	(0.125)	(0.127)
Observations	102	100	100	97	97
R-squared	0.064	0.101	0.219	0.338	0.454
Continent Fixed Effects			Yes		Yes

Table 3. Results from OLS regression of WVS average country-level Happiness on Hunting or Gathering Predominant Ancestry (Column 1) as well as including contemporary and historical controls (Columns 2-5). Robust standard errors in parentheses. *** p < 0.01, ** p < 0.05, * p < 0.1.

Results for life satisfaction were qualitatively similar to the results on happiness. The fraction of the population with ancestry from gathering-predominant societies is positively associated with country-level life satisfaction, controlling for contemporary income per capita (beta = 7.74; Standard Error (SE) = 4.5, R^2^ = 33.0%, p < 0.1; Table 4, Column 2). The fraction of population ancestry from gatherer-predominant populations is positively associated with country-level life satisfaction with or without including controls for GDP per capita, historical controls, or continent fixed effects (Table 4; Columns 2–5). Results for life satisfaction and hunting, as well as for hunting or gathering ancestry, were qualitatively similar to those for happiness, and are reported in Supporting Information Tables S1 and S2 in S1 File. Inclusion of Fishing predominant ancestry with hunting and gathering ancestry did not change results qualitatively, as reported in Supporting Information Tables S3-S5 in S1 File.

**Table 4:** Gathering Predominant Ancestry Fraction and Country-Level Life Satisfaction.

	(1)	(2)	(3)	(4)	(5)
	Life Satisfaction				
Gathering Predominant Ancestry	8.444**	7.748*	4.722*	10.67**	9.070***
	(4.199)	(4.498)	(2.773)	(4.889)	(3.016)
Log GDP Per Capita 2019 (WDI)		0.0227***	0.0249***	0.0257***	0.0293***
		(0.00310)	(0.00437)	(0.00444)	(0.00454)
Average Level of Political Hierarchy				0.0446***	0.0421***
				(0.0147)	(0.0154)
Average Settlement Complexity				−0.00420	−0.00488
				(0.00715)	(0.00727)
Patrilineal Descent				0.0933***	0.108***
				(0.0290)	(0.0287)
Matrilineal Descent				0.0110	0.0990*
				(0.0630)	(0.0538)
Polygynous				−0.0823	−0.00868
				(0.0585)	(0.0537)
Plough Use				−0.168***	−0.152***
				(0.0338)	(0.0339)
Kinship Score				−0.141***	−0.120***
				(0.0404)	(0.0391)
Constant	0.639***	0.219***	0.165**	0.245**	0.104
	(0.0109)	(0.0617)	(0.0708)	(0.111)	(0.104)
Observations	102	100	100	97	97
R-squared	0.022	0.330	0.480	0.492	0.634
Continent Fixed Effects			Yes		Yes

Table 4. Results from OLS regression of WVS average country-level Life Satisfaction on Gathering Predominant Ancestry (Column 1) as well as including contemporary and historical controls (Columns 2-5). Robust standard errors in parentheses. *** p < 0.01, ** p < 0.05, * p < 0.1.

## Discussion

There appears to be a statistically significant association between the fraction of ancestry from gatherer-predominant societies and contemporary average country-level happiness. The relationship is robust to a set of commonly used contemporary and historical controls, as well as continent fixed effects. The results also suggest a smaller effect for the influence on hunter-predominant ancestry, although these results were not fully robust to the inclusion of historical controls and continent effects. The findings are also notable given the limited quantitative contribution of hunting and gathering predominant societies, accounting for 178 of 1265 societies in the Ethnographic Atlas.

There are several potential explanations for these results. One important explanation is that the finding could be due to omitted variable bias, a threat to any study using associative techniques. Although the Gatherer results are robust to an extensive list of controls, those including hunting are less robust with inclusion of continent controls. It is also notable that the baseline fractions of hunter and gatherer ancestry in most countries are quite small. However, it is possible that the country-level cultural influences of this ancestry extend beyond the estimated quantitative fraction. While we caution against any causal interpretation of these results, several features of the analysis support a real association between these variables. These factors include the historical nature of the predictor of interest, with ancestral fraction contributions set in time well before the estimation of contemporary happiness rates. Additionally, the statistically and economically significant positive relationship remained strong across specifications, including uncontrolled regression as well as OLS regression with rich sets of historical controls, contemporary GDP.

The possibility of a relationship between gatherer ancestry and societal happiness is consistent with studies of wellbeing in contemporary hunter-gatherer societies [10,44,45]. Franckowiak and colleagues (2021) find subjective happiness among the Hadza compares favorably to a sample of Polish individuals, while Reyes-Garcia et al 2021 find notably high and seasonally stable levels of wellbeing among their study populations from three small-scale societies [16,37]. Theorists from Thoreau to Weber have posited that detachment from nature has had negative implications for the human experience, such as separation from natural cycles of life [46,47]. The growing body of evidence for nature exposure and social rhythm therapy in various psychiatric disorders attests to the salience of this concept to human affective wellbeing [48,49]. Another potential mechanism, the argument of Rousseau’s famous 1761 discourse on the origin of inequality, is that human hierarchies are responsible for a large fraction of human unhappiness, and emotional wellbeing may have been higher in ancestral hunter-gatherer societies due to reduced class stratification [9]. This mechanism could be driven by either material inequality or formation of status hierarchy, a distinction beyond the scope of the analysis in this paper but indicated for future study [50,51]. Of note, average ancestral level of political hierarchy and average ancestral settlement complexity were included as controls in the specification, and were not significant.

There are several limitations to this study. One important limitation is the use of the *Ethnologue* approach to linking ancestral societal features to modern populations. In the approach, a country’s ancestral representation in the Murdock Atlas is estimated using the *Ethnologue* language atlas. With the prevalence of language as the basis of mapping societies in the Murdock atlas, there is much additional ancestral richness and heterogeneity that is not incorporated in this approach. This critique has been noted in several key papers in historical economics, which nevertheless utilize the *Ethnologue* approach as the most effective known method for linking the Murdock Atlas to modern nation states [26,28–30,52]. Another important limitation is the associative nature of the findings as discussed above, and caution should be used in applying a causal interpretation. Additionally, the World Values Survey data covers only 104 countries, omitting variation from dozens of countries globally [41]. Further investigation could link micro-level analyses of wellbeing to the Ethnographic Atlas, or identify ecological instruments for gathering ancestry to improve identification.

## Conclusion

Contemporary techniques and data availability in historical economics have indicated a positive, economically significant relationship between the fraction of ancestry from gatherer societies and contemporary average subjective wellbeing. While additional work is necessary to confirm this relationship, the study finds that ancestral social organization may contribute meaningfully to modern population-level wellbeing, and that these relationships can be characterized using techniques from historical economics. Given the limitations of economic growth in improving happiness, and negative externalities including global warming, gatherer ancestry may hold wisdom for population wellbeing worth further exploration.

## Supporting information

S1 File Supporting Information. Tables S3-S5 are included in the Supporting Information file.
(DOCX)
