# Supplementary material for: Gatherer ancestry associated with national happiness
Source: PLoS One. 2026 Jan 21;21(1):e0336161. doi: 10.1371/journal.pone.0336161 (PMC12822993; doi:10.1371/journal.pone.0336161)
Supplement: S1 File — Tables S3-S5 are included in the Supporting Information file. (DOCX) [file pone.0336161.s001.docx]

**Supporting Information: Gatherer Ancestry Associated with National Happiness**

Matthew Basilico MD PhD

**Table S1: Hunting Predominant Ancestry Fraction and Country-Level Life Satisfaction**

|  | (1) | (2) | (3) | (4) | (5) |
| --- | --- | --- | --- | --- | --- |
|  | Life Satisfaction: general satisfaction with one's life | | | | |
| Hunting Predominant | 7.378*** | 6.165*** | -1.222 | 3.985*** | -3.168 |
|  | (1.491) | (0.897) | (2.352) | (1.135) | (2.200) |
| Log GDP Per Capita 2019 (WDI) |  | 0.0224*** | 0.0250*** | 0.0255*** | 0.0289*** |
|  |  | (0.00306) | (0.00437) | (0.00443) | (0.00448) |
| Average Level of Political Hierarchy |  |  |  | 0.0368** | 0.0417*** |
|  |  |  |  | (0.0148) | (0.0157) |
| Average Settlement Complexity |  |  |  | -0.00344 | -0.00394 |
|  |  |  |  | (0.00697) | (0.00723) |
| Patrilineal Descent |  |  |  | 0.0808*** | 0.101*** |
|  |  |  |  | (0.0285) | (0.0285) |
| Matrilineal Descent |  |  |  | -0.00619 | 0.0926* |
|  |  |  |  | (0.0601) | (0.0511) |
| Polygynous |  |  |  | -0.0723 | 0.00607 |
|  |  |  |  | (0.0573) | (0.0527) |
| Plough Use |  |  |  | -0.158*** | -0.146*** |
|  |  |  |  | (0.0332) | (0.0328) |
| Kinship Score |  |  |  | -0.128*** | -0.127*** |
|  |  |  |  | (0.0401) | (0.0385) |
| Constant | 0.640*** | 0.225*** | 0.165** | 0.259** | 0.112 |
|  | (0.0107) | (0.0609) | (0.0707) | (0.111) | (0.105) |
| Observations | 102 | 100 | 100 | 97 | 97 |
| R-squared | 0.029 | 0.334 | 0.475 | 0.471 | 0.620 |
| Continent FE |  |  | YES |  | YES |

Table S1: Results from OLS regression of WVS average country-level Life Satisfaction on Hunting Predominant Ancestry (Column 1) as well as including contemporary and historical controls (Columns 2-5). Robust standard errors in parentheses. *** p<0.01, ** p<0.05, * p<0.1

**Table S2: Hunting or Gathering Predominant Ancestry Fraction and Country-Level Life Satisfaction**

|  | (1) | (2) | (3) | (4) | (5) |
| --- | --- | --- | --- | --- | --- |
|  | Life Satisfaction: general satisfaction with one's life | | | | |
| Hunting or Gathering Predominant | 7.293*** | 6.273*** | 0.893 | 5.849*** | 1.064 |
|  | (1.767) | (1.480) | (1.360) | (1.984) | (2.134) |
| Log GDP Per Capita 2019 (WDI) |  | 0.0224*** | 0.0248*** | 0.0260*** | 0.0287*** |
|  |  | (0.00309) | (0.00436) | (0.00443) | (0.00448) |
| Average Level of Political Hierarchy |  |  |  | 0.0386*** | 0.0395** |
|  |  |  |  | (0.0141) | (0.0154) |
| Average Settlement Complexity |  |  |  | -0.00429 | -0.00425 |
|  |  |  |  | (0.00699) | (0.00723) |
| Patrilineal Descent |  |  |  | 0.0855*** | 0.0989*** |
|  |  |  |  | (0.0279) | (0.0281) |
| Matrilineal Descent |  |  |  | 0.00485 | 0.0892* |
|  |  |  |  | (0.0615) | (0.0513) |
| Polygynous |  |  |  | -0.0774 | 0.00293 |
|  |  |  |  | (0.0564) | (0.0529) |
| Plough Use |  |  |  | -0.158*** | -0.144*** |
|  |  |  |  | (0.0325) | (0.0326) |
| Kinship Score |  |  |  | -0.124*** | -0.119*** |
|  |  |  |  | (0.0393) | (0.0382) |
| Constant | 0.637*** | 0.222*** | 0.167** | 0.244** | 0.117 |
|  | (0.0109) | (0.0615) | (0.0705) | (0.111) | (0.104) |
| Observations | 102 | 100 | 100 | 97 | 97 |
| R-squared | 0.047 | 0.348 | 0.475 | 0.491 | 0.617 |
| Continent FE |  |  | YES |  | YES |

Table S2: Results from OLS regression of WVS average country-level Life Satisfaction on Hunting or Gathering Predominant Ancestry (Column 1) as well as including contemporary and historical controls (Columns 2-5). Robust standard errors in parentheses. *** p<0.01, ** p<0.05, * p<0.1

**Table S3: Fishing Predominant Ancestry Fraction and Country-Level Happiness**

|  | (1) | (2) | (3) | (4) | (5) |
| --- | --- | --- | --- | --- | --- |
|  | Happiness | | | | |
| Fishing Predominant | 3.671** | 3.221* | 0.482 | 1.913 | 0.295 |
|  | (1.445) | (1.917) | (1.999) | (2.009) | (2.499) |
| Log GDP Per Capita 2019 (WDI) |  | 0.00732** | 0.0136*** | 0.0137*** | 0.0177*** |
|  |  | (0.00310) | (0.00409) | (0.00437) | (0.00409) |
| Average Level of Political Hierarchy |  |  |  | 0.0165 | 0.0220 |
|  |  |  |  | (0.0156) | (0.0144) |
| Average Settlement Complexity |  |  |  | 0.00334 | 0.00582 |
|  |  |  |  | (0.00715) | (0.00816) |
| Patrilineal Descent |  |  |  | 0.00954 | 0.0115 |
|  |  |  |  | (0.0344) | (0.0278) |
| Matrilineal Descent |  |  |  | -0.142* | -0.0924 |
|  |  |  |  | (0.0828) | (0.0901) |
| Polygynous |  |  |  | 0.0228 | 0.0832 |
|  |  |  |  | (0.0442) | (0.0634) |
| Plough Use |  |  |  | -0.160*** | -0.158*** |
|  |  |  |  | (0.0335) | (0.0332) |
| Kinship Score |  |  |  | -0.0382 | -0.0460 |
|  |  |  |  | (0.0489) | (0.0400) |
| Constant | 0.700*** | 0.564*** | 0.473*** | 0.523*** | 0.390*** |
|  | (0.00927) | (0.0617) | (0.0664) | (0.126) | (0.128) |
| Observations | 102 | 100 | 100 | 97 | 97 |
| R-squared | 0.031 | 0.064 | 0.211 | 0.304 | 0.447 |
| Continent FE |  |  | YES |  | YES |

Table S2: Results from OLS regression of WVS average country-level Happiness on Fishing Predominant Ancestry (Column 1) as well as including contemporary and historical controls (Columns 2-5). Robust standard errors in parentheses. *** p<0.01, ** p<0.05, * p<0.1

**Table S4: Fishing, Hunting or Gathering Predominant Ancestry Fraction and Country-Level Happiness**

|  | (1) | (2) | (3) | (4) | (5) |
| --- | --- | --- | --- | --- | --- |
|  | Happiness | | | | |
| Hunting, Gathering or Fishing Predominant | 7.225*** | 6.618*** | 3.056 | 5.882** | 2.790 |
|  | (2.671) | (2.469) | (2.307) | (2.735) | (2.788) |
| Log GDP Per Capita 2019 (WDI) |  | 0.00721** | 0.0134*** | 0.0144*** | 0.0178*** |
|  |  | (0.00311) | (0.00410) | (0.00437) | (0.00409) |
| Average Level of Political Hierarchy |  |  |  | 0.0158 | 0.0211 |
|  |  |  |  | (0.0148) | (0.0140) |
| Average Settlement Complexity |  |  |  | 0.00262 | 0.00549 |
|  |  |  |  | (0.00708) | (0.00815) |
| Patrilineal Descent |  |  |  | 0.0122 | 0.0126 |
|  |  |  |  | (0.0338) | (0.0273) |
| Matrilineal Descent |  |  |  | -0.135* | -0.0927 |
|  |  |  |  | (0.0805) | (0.0883) |
| Polygynous |  |  |  | 0.0159 | 0.0778 |
|  |  |  |  | (0.0450) | (0.0642) |
| Plough Use |  |  |  | -0.159*** | -0.159*** |
|  |  |  |  | (0.0326) | (0.0333) |
| Kinship Score |  |  |  | -0.0275 | -0.0410 |
|  |  |  |  | (0.0489) | (0.0401) |
| Constant | 0.697*** | 0.563*** | 0.475*** | 0.506*** | 0.390*** |
|  | (0.00928) | (0.0619) | (0.0665) | (0.125) | (0.127) |
| Observations | 102 | 100 | 100 | 97 | 97 |
| R-squared | 0.064 | 0.101 | 0.219 | 0.338 | 0.454 |
| Continent FE |  |  | YES |  | YES |

Table S4: Results from OLS regression of WVS average country-level Happiness on Fishing, Hunting or Gathering Predominant Ancestry (Column 1) as well as including contemporary and historical controls (Columns 2-5). Robust standard errors in parentheses. *** p<0.01, ** p<0.05, * p<0.1

**Table S5: Fishing, Hunting or Gathering Predominant Ancestry Fraction and Country-Level Life Satisfaction**

|  | (1) | (2) | (3) | (4) | (5) |
| --- | --- | --- | --- | --- | --- |
|  | Life Satisfaction | | | | |
| Hunting, Gathering or Fishing Predominant | 7.293*** | 6.273*** | 0.893 | 5.849*** | 1.064 |
|  | (1.767) | (1.480) | (1.360) | (1.984) | (2.134) |
| Log GDP Per Capita 2019 (WDI) |  | 0.0224*** | 0.0248*** | 0.0260*** | 0.0287*** |
|  |  | (0.00309) | (0.00436) | (0.00443) | (0.00448) |
| Average Level of Political Hierarchy |  |  |  | 0.0386*** | 0.0395** |
|  |  |  |  | (0.0141) | (0.0154) |
| Average Settlement Complexity |  |  |  | -0.00429 | -0.00425 |
|  |  |  |  | (0.00699) | (0.00723) |
| Patrilineal Descent |  |  |  | 0.0855*** | 0.0989*** |
|  |  |  |  | (0.0279) | (0.0281) |
| Matrilineal Descent |  |  |  | 0.00485 | 0.0892* |
|  |  |  |  | (0.0615) | (0.0513) |
| Polygynous |  |  |  | -0.0774 | 0.00293 |
|  |  |  |  | (0.0564) | (0.0529) |
| Plough Use |  |  |  | -0.158*** | -0.144*** |
|  |  |  |  | (0.0325) | (0.0326) |
| Kinship Score |  |  |  | -0.124*** | -0.119*** |
|  |  |  |  | (0.0393) | (0.0382) |
| Constant | 0.637*** | 0.222*** | 0.167** | 0.244** | 0.117 |
|  | (0.0109) | (0.0615) | (0.0705) | (0.111) | (0.104) |
| Observations | 102 | 100 | 100 | 97 | 97 |
| R-squared | 0.047 | 0.348 | 0.475 | 0.491 | 0.617 |
| Continent FE |  |  | YES |  | YES |

Table S5: Results from OLS regression of WVS average country-level Life Satisfaction on Fishing, Hunting or Gathering Predominant Ancestry (Column 1) as well as including contemporary and historical controls (Columns 2-5). Robust standard errors in parentheses. *** p<0.01, ** p<0.05, * p<0.1
