# Supplementary material for: Gatherer ancestry associated with national happiness
Source: PLoS One. 2026 Jan 21;21(1):e0336161. doi: 10.1371/journal.pone.0336161 (PMC12822993; doi:10.1371/journal.pone.0336161)
Supplement: Attachment — Submitted filename: Response to Reviewers.pdf [file pone.0336161.s003.pdf]

HARVARD UNIVERSITY FACULTY OF ARTS & SCIENCES
DEPARTMENT OF ECONOMICS

1805 Cambridge Street
Cambridge, MA 02138
Tel. (617) 775-6992
matthew_basilico@hms.harvard.edu

October 21, 2025

RE: Response to Reviewers

Dear Professor Barkai,

Thank you very much for these detailed and helpful comments. I am extremely appreciative of the time and thoughtfulness of your efforts and those of the reviewers.

I have updated the draft below based on their comments. I have also significantly augmented the coverage of previous related studies per your feedback.

I have included below the reviewers' comments with my responses to each comment in red text.

Thank you again for your helpful feedback, and very happy to rework as necessary so that this finding may be available in the literature.

Thank you, and all best wishes,

Matthew Basilico MD, PhD
Assistant Professor of Psychiatry, Harvard Medical School
Associate Psychiatrist, Mass General Brigham McLean Hospital
Lecturer, Department of Economics, Harvard University Faculty of Arts and Sciences
Lecturer, Department of Global Health and Social Medicine, Harvard Medical School
matthew_basilico@hms.harvard.edu
(617) 775 6992

1. Please ensure that your manuscript meets PLOS ONE's style requirements, including those for file naming. The PLOS ONE style templates can be found at https://journals.plos.org/plosone/s/file?id=wjVg/PLOSOne_formatting_sample_main_body.pdf and https://journals.plos.org/plosone/s/file?id=ba62/PLOSOne_formatting_sample_title_authors_affiliations.pdf

I have reviewed the style requirements, including file naming, and to my understanding this draft is consistent with the requirements. I am very happy to make any additional adjustments if indicated.

2. We note that your Data Availability Statement is currently as follows: [All relevant data are within the manuscript and its Supporting Information files.]

Please confirm at this time whether or not your submission contains all raw data required to replicate the results of your study. Authors must share the “minimal data set” for their submission. PLOS defines the minimal data set to consist of the data required to replicate all study findings reported in the article, as well as related metadata and methods (https://journals.plos.org/plosone/s/data-availability#loc-minimal-data-set-definition).

For example, authors should submit the following data:

- The values behind the means, standard deviations and other measures reported;
- The values used to build graphs;
- The points extracted from images for analysis.

Authors do not need to submit their entire data set if only a portion of the data was used in the reported study.

All the data used in this study is publicaly available, from the four following data sources: the World Bank World Development Indicators, the World Values Survey, the supporting files of Giuliano and Nunn 2018 and supporting files of Enke 2019.

However, I have now also taken the additional step of posting the merged dataset and STATA code for production of all tables in the analyses in github: https://github.com/mfbasilico/GatheringHappiness. The posted code reproduces all tables.

If your submission does not contain these data, please either upload them as Supporting Information files or deposit them to a stable, public repository and provide us with the relevant URLs, DOIs, or accession numbers. For a list of recommended repositories, please see https://journals.plos.org/plosone/s/recommended-repositories.

If there are ethical or legal restrictions on sharing a de-identified data set, please explain them in detail (e.g., data contain potentially sensitive information, data are owned by a third-party organization, etc.) and who has imposed them (e.g., an ethics committee). Please also provide contact information for a data access committee, ethics committee, or other institutional body to which data requests may be sent. If data are owned by a third party, please indicate how others may request data access.
3. We note that Figure 1 in your submission contain [map/satellite] images which may be copyrighted. All PLOS content is published under the Creative Commons Attribution License (CC BY 4.0), which means that the manuscript, images, and Supporting Information files will be freely available online, and any

third party is permitted to access, download, copy, distribute, and use these materials in any way, even commercially, with proper attribution. For these reasons, we cannot publish previously copyrighted maps or satellite images created using proprietary data, such as Google software (Google Maps, Street View, and Earth). For more information, see our copyright guidelines: http://journals.plos.org/plosone/s/licenses-and-copyright.

We require you to either (1) present written permission from the copyright holder to publish these figures specifically under the CC BY 4.0 license, or (2) remove the figures from your submission:

1. You may seek permission from the original copyright holder of Figure 1 to publish the content specifically under the CC BY 4.0 license.

We recommend that you contact the original copyright holder with the Content Permission Form (http://journals.plos.org/plosone/s/file?id=7c09/content-permission-form.pdf) and the following text:

“I request permission for the open-access journal PLOS ONE to publish XXX under the Creative Commons Attribution License (CCAL) CC BY 4.0 (http://creativecommons.org/licenses/by/4.0/). Please be aware that this license allows unrestricted use and distribution, even commercially, by third parties. Please reply and provide explicit written permission to publish XXX under a CC BY license and complete the attached form.”

Please upload the completed Content Permission Form or other proof of granted permissions as an ""Other"" file with your submission.

In the figure caption of the copyrighted figure, please include the following text: “Reprinted from [ref] under a CC BY license, with permission from [name of publisher], original copyright [original copyright year].”

2. If you are unable to obtain permission from the original copyright holder to publish these figures under the CC BY 4.0 license or if the copyright holder’s requirements are incompatible with the CC BY 4.0 license, please either i) remove the figure or ii) supply a replacement figure that complies with the CC BY 4.0 license. Please check copyright information on all replacement figures and update the figure caption with source information. If applicable, please specify in the figure caption text when a figure is similar but not identical to the original image and is therefore for illustrative purposes only.

The following resources for replacing copyrighted map figures may be helpful:

USGS National Map Viewer (public domain): http://viewer.nationalmap.gov/viewer/

The Gateway to Astronaut Photography of Earth (public domain): http://eol.jsc.nasa.gov/sseop/clickmap/

Maps at the CIA (public domain): https://www.cia.gov/library/publications/the-world-factbook/index.html and https://www.cia.gov/library/publications/cia-maps-publications/index.html

NASA Earth Observatory (public domain): http://earthobservatory.nasa.gov/

Landsat: http://landsat.visibleearth.nasa.gov/

USGS EROS (Earth Resources Observatory and Science (EROS) Center) (public domain): http://eros.usgs.gov/#

Natural Earth (public domain): http://www.naturalearthdata.com/

Thank you very much for this guidance. After reflection including Reviewer 2 Comment #7 as well as copyright limitations, I am in agreement that the figure does not add sufficiently to the analysis, and that these results are available elsewhere. I have included an additional table in the manuscript from the helpful suggestion regarding Life Satisfaction, and instead of a map I have included summary statistics within the text on the prevalence and variance of hunter and gatherer ancestry using the Ethnologue approach as suggested by this comment.

4. If the reviewer comments include a recommendation to cite specific previously published works, please review and evaluate these publications to determine whether they are relevant and should be cited. There is no requirement to cite these works unless the editor has indicated otherwise.

Thank you very much for this comment. The updated manuscript includes an expanded literature review which touches on additional themes mentioned in the reviewers and editors, including wellbeing in small-scale societies, limitations to the Ethnologue approach, and the citations suggested by Reviewer 2 in Comment #5.

[Note: HTML markup is below. Please do not edit.]

Reviewers' comments:

Reviewer's Responses to Questions
Comments to the Author

1. Is the manuscript technically sound, and do the data support the conclusions?

The manuscript must describe a technically sound piece of scientific research with data that supports the conclusions. Experiments must have been conducted rigorously, with appropriate controls, replication, and sample sizes. The conclusions must be drawn appropriately based on the data presented.
Reviewer #1: Yes
Reviewer #2: Partly

---

2. Has the statistical analysis been performed appropriately and rigorously?
Reviewer #1: I Don't Know
Reviewer #2: Yes

As mentioned above, I have included data and STATA code used for each of the tables publicly on github. Of note, the standard errors in columns 4 & 5 of Tables 1-3 have been updated to heteroskedasticity-robust as originally indicated in the methods section.

---

3. Have the authors made all data underlying the findings in their manuscript fully available?

The PLOS Data policy requires authors to make all data underlying the findings described in their manuscript fully available without restriction, with rare exception (please refer to the Data Availability Statement in the manuscript PDF file). The data should be provided as part of the manuscript or its supporting information, or deposited to a public repository. For example, in addition to summary statistics, the data points behind means, medians and variance measures should be available. If there are restrictions on publicly sharing data—e.g. participant privacy or use of data from a third party—those must be specified.
Reviewer #1: Yes
Reviewer #2: No

As mentioned above, all data used in the analysis is available from 4 publicly available datasources (World Bank WDI, World Values Survey, Enke 2019 and Giuliano-Nunn 2018). I have taken the additional step of posting merged dataset with all data elements used in the analysis, and code for the tables.

4. Is the manuscript presented in an intelligible fashion and written in standard English?

PLOS ONE does not copyedit accepted manuscripts, so the language in submitted articles must be clear, correct, and unambiguous. Any typographical or grammatical errors should be corrected at revision, so please note any specific errors here.
Reviewer #1: Yes
Reviewer #2: Yes

---

5. Review Comments to the Author

Please use the space provided to explain your answers to the questions above. You may also include additional comments for the author, including concerns about dual publication, research ethics, or publication ethics. (Please upload your review as an attachment if it exceeds 20,000 characters)
Reviewer #1: This is a very significant article, and with a few additions and some clearer wording, should definitely be published

The paper is about two related, but distinct, hypothesized phenomena. The first is that there exist "links between hunter-gatherer economic organization and emotional wellbeing". The second is that these links may be passed on to subsequent generations that have transitioned to non-hunter-gatherer economies "through transgenerational cultural transmission". From the author's conclusion, it is clear that the second of these phenomena is what the paper focusses on and hopes to demonstrate. This is confirmed in the first sentence in the methodology section, which states: "This study examines how the variance in measured average population-level happiness varies with the fraction of ancestry from hunter-predominant, gather-predominant, or agricultural-predominant societies."

However, the author needs to signal that these are two rather different phenomena, particularly in terms of what might account for them. In the case of the first hypothesis, the author briefly suggests possible explanations: that "forms of economic organization more closely tied to rhythms of natural environmental cycles contained advantages for emotional wellbeing", and also that emotional wellbeing is related to the relative lack of inequality and "a lack of class stratification" among hunter-gatherers.

Where the author refers to the relationship between happiness and low levels of inequality, some reference is needed to the distinction between material inequality and inequality based on the existence of a status hierarchy, as these may not coincide.

Thank you very much for this thoughtful comment. I have provided additional discussion on the distinction between material inequality and status hierarchy, and how each could be relevant to the results of the analysis.

In the case of the second hypothesis, the author leaves several issues only briefly examined, and in particular when. why and how the cultural transmission of the ancestral benefits for hunter-gatherer emotional well-being persist or do not persist in descendant generations. Much depends on the author's reliance on a single 2018 source, Giuliano P, Nunn N. Ancestral Characteristics of Modern Populations. Economic History of Developing Regions. Part of the issue here is in the method to distinguish between a population with an ancestry from hunter-gatherer societies, and those without such an ancestry. This distinction is made on the basis of language, also implying that this is the mode of cultural transmission. According to Google Scholar, the Giuliano and Nunn article has been cited no less than 117 times. Given

its key place in the author's argument, I would have expected some discussion of any issues raised that are relevant to his hypothesis in this subsequent literature.

Thank you very much for this important comment. I have expanded the discussion of critiques of the *Ethnologue* approach within the Introduction and Discussion sections, within the section on study limitations. This approach has been used in dozens of papers within the historical economics literature; however, as you note there are important limitations, notably the assignment of a language group to an observation in the ethnographic atlas, and the use of language as the sole connection to ancestry in contemporary nation-states.

In addition, I found that in some places the wording was either a bit awkward, ungrammatical, or difficult to understand. As examples, I note the following:

"and appears to have intellectual the culture of Anglo-American analytic philosophy, including the notion of preference optimization dating to Jeremy Bentham."

"Social theorists including Freud, Durkheim and Rousseau identified potential links between hunter-gatherer economic organization and emotional wellbeing that would not be well captured by a utility from consumption framework."

"caution should be used in applying a causal."

Thank you very much for these helpful examples. I edited each of areas mentioned to use clearer wording. I have also reviewed the manuscript for similar instances where the language could be clearer and adjusted accordingly.

Reviewer #2: This article combines a previously-published method linking the geographical distribution of ethnic ancestry, via language groups and pre-industrial ethnographic records, with the World Values Survey life satisfaction scores. The main finding is a statistically-significant, positive relationship between the estimated fraction of Gatherer ancestry and life satisfaction, at the national level. I think this is an interesting and important finding, worthy of publication. However I think the article is not sufficiently developed at present, and there are a number of aspects that need to be strengthened so that the analyses are performed to a high technical standard and the conclusions adequately supported.

1. I think it's important to expand the discussion of the ethnologue approach. I know the linking to the ethnography is not the new contribution of this work, but I was surprised that the original references for the method did not seem to address this aspect. Is the United States linked, in this analysis, to an English heritage because that's the predominant language for the country? If so, how does this affect countries that strongly enforced national languages in the 19th century, like France? A bit of discussion about this seems pertinent - there might be systematic underestimates of ancestry of groups that were coerced to speak a dominant language rather than maintaining their traditional language. I think it's quite important for the conclusions and needs to be discussed, particularly given that prior research has shown that immigrants tend to shift their life satisfaction toward their new country, even within a given lifetime (e.g. Helliwell et al., International Migration and World Happiness, World Happiness Report 2018).

Thank you very much for this helpful comment. As noted above in my comment to Reviewer #1 who recognized a similar important issue, I have expanded my discussion on the use of the *Ethnologue* approach under the Introduction and Limitations sections. Despite its role as a standard-bearer in this

style of analysis in cultural persistence studies within historical economics, there are a few key limitations with the approach including those noted here, and I have strived to be fully transparent.

2. It's not clear to me that the binary distinctions of 'gathering predominant' vs. 'hunting predominant' is the best way to analyze the atlas data. Forager lifeways were diverse, and usually involved a combination of hunting, fishing and gathering in variable proportions (see e.g. Kelly, 2013, Lifeways of Hunter Gatherers). It would therefore be surprising that the hunting-predominant societies were not also doing a lot of gathering (and most were probably fishing too). I see that the Atlas includes a column that actually assesses all forms of subsistence - could this be used instead of the simple binary distinctions?

Thank you very much for this comment. Variable 42 in the Ethnographic Atlas assigns each society a value based on the predominant mode of subsistence. As discussed in the reply to Comment #6 and now in the Methods section, the predominant mode was agriculture (intensive, extensive, or type unknown). Per your suggestion, I have also run the analysis on Fishing predominant societies, as well as examined the fraction of ancestry from all 3 categories (Fishing, Hunting or Gathering predominant: see Appendix Tables S3 and S5). The results are qualitatively similar to the results for Hunting and Gathering predominant societies.

3. I feel the paper needs some more substantive thought into exactly how the ancestral populations may be connected to average national life satisfaction. I presume that the fractions of modern populations that are linked to either gathering or hunting are mostly very small? (A map would be very helpful, to show the % of each ancestry by country.) If so, how would it be that these small populations manage to exert significant influence on the entire national populations? There is some literature on South American societies that might be useful for this, such as regarding the Buen Vivir idea (Villalba, Buen Vivir vs. Development: a paradigm shift in the Andes? Third World Quarterly, 2014). Related to this, it also might be helpful to discuss the fact that really this is getting at populations with recent hunting and gathering ancestry, since all humans are descended 100% from hunters and gatherers if one goes back far enough in time.

Thank you very much for this comment, and it is one which has prompted a lot of thinking on my own end.

First to the more practical component of the comment: I have included summary statistics regarding the average fractions and variances of hunter or gather predominant, hunter-predominant and gatherer-predominant ancestries. Indeed, as you note, the fractions are quite small on average but do have high variation.

Second, your comment that "all humans are descended 100% from hunters and gatherers if one goes back far enough" has led me to rework components of the analysis and interpretation. Indeed, from this helpful vantage point, the interpretation may ultimately involve how transgenerational exposure to non-subsistence lifestyles leads to a worsening of human wellbeing. While the available statistical analysis in would be similar under each interpretation (preservation of ancestral subsistent culture, versus exposure to non-subsistence cultures), the alternative framing provided can help link to future analysis. The Introduction has been expanded to address this comment directly.

4. I think it would also be worthwhile comparing with the Gallup world poll results. There is some important nuance between how people answer the Cantril ladder vs. life satisfaction questions (Nilsson, The Cantril Ladder elicits thoughts about power and wealth, Scientific Reports

2024 https://doi.org/10.1038/s41598-024-52939-y), so I don't think it would undermine the results here if the Gallup results were not similarly correlated with hunter-gatherer ancestry. Whatever it shows, it would provide useful additional insight.

Thank you very much for this very helpful suggestion. Indeed, the World Values Survey itself contains a Life Satisfaction measure distinct from the affective Happiness measure, with construct validity similar to the Cantril Ladder. I have included the results in a new Table 4 of the analysis, with interpretation, with additional tables in the appendix. Indeed, the results are similar qualitatively to the affective/happiness results, indicating a similar overall pattern. A quick note on Gallup versus WVS: the Gallup data for this analysis requires a subscription, versus the WVS data which is publicly available. I would not have reason to believe that the results would be substantively different for the purposes of this analysis, and use of the WVS data allows preservation of the open access spirit of the investigation.

5. There are also some additional references that may prove helpful. The works by Minarro (Miñarro et al., Happy without money: Minimally monetized societies can exhibit high subjective well-being. PLoS ONE 2021 https://doi.org/10.1371/journal.pone.0244569) and Galbraith (Galbraith et al., High life satisfaction reported among small- scale societies with low incomes, PNAS 2024 https://doi.org/10.1073/pnas.2311703121) seem particularly relevant, and would tend to support the findings here with a more direct, though more locally-focused method.

Thank you very much for these suggestions. These references were reviewed and are included in addition to an enhancement of the Introduction as noted above, in addition to several other sources.

6. The discussion states that 'findings are also notable the limited quantitative contribution of hunting and gathering predominant societies, accounting for 180 of 1265 societies in the Ethnographic Atlas.' So what were the other 1085 societies doing for subsistence?

Thank you very much for this comment. I have looked to incorporate the substance of this reflection into the response to Comment #3 regarding alternative perspective on the results. Reviewing Ethnographic Atlas variable 42, which assigns societies based on "dominant mode of subsistence": Agriculture (extensive, intensive, or type unknown) and Pastoralism accounted for the vast majority of societies. The methods section now lists each of the categories in v42, as well as counts all other categories in the dataset, demonstrating the agricultural predominance.

This is a reflection of the composition of the Ethnographic Atlas (compiled by George Murdock using first known ethnographic analyses), and hence typically reflecting several hundred to several thousand years of social organization dating up to the 20th century. This period is associated with the evolution of substantial human cultural heterogeneity, as indicated in work by Joseph Henrich and others. As you noted in Comment #3, going back far enough all humans are hunter-gatherers. The coding here reflects a measure (not without flaws, but thought to be best available) of economic organization for numerous generations before the 20th century, capturing one important influence on cultural norms.

7. The figure does not seem appropriate. This figure simply shows the previously reported WVS life satisfaction score by country, without adding anything related to the work at present. I think it would be far more useful to include a figure of the hunter and gatherer ancestry fractions by country, or similar. Perhaps the three could be shown as adjacent panels - each of the two ancestries, and the life satisfaction?

Thank you very much for this comment. As noted above, after reflection, as well as copyright restrictions, I have opted to remove this figure from the updated draft. Instead, I have included summary statistics regarding the fractions of hunter-gather predominant, hunter-predominant, and gather-predominant populations in the results section, and included a new Table 4, as well as appendix tables, with results on Life Satisfaction.

Again I think this is potentially very valuable work and hope that these comments can help to strengthen it.

Thank you!
