# Supplementary material for: Gatherer ancestry associated with national happiness
Source: PLoS One. 2026 Jan 21;21(1):e0336161. doi: 10.1371/journal.pone.0336161 (PMC12822993; doi:10.1371/journal.pone.0336161)
Supplement: Peer review history

# Peer review: Decision Letter 0

**DOI:** 10.1371/journal.pone.0336161.r001

**Authors:** Ran Barkai

8 Sep 2025

Dear Dr. Basilico,

Thank you for submitting your manuscript to PLOS ONE. After careful consideration, we feel that it has merit but does not fully meet PLOS ONE’s publication criteria as it currently stands. Therefore, we invite you to submit a revised version of the manuscript that addresses the points raised during the review process.

Please submit your revised manuscript by Oct 23 2025 11:59PM. If you will need more time than this to complete your revisions, please reply to this message or contact the journal office at plosone@plos.org . When you're ready to submit your revision, log on to https://www.editorialmanager.com/pone/ and select the 'Submissions Needing Revision' folder to locate your manuscript file.

- A rebuttal letter that responds to each point raised by the academic editor and reviewer(s). You should upload this letter as a separate file labeled 'Response to Reviewers'.
- A marked-up copy of your manuscript that highlights changes made to the original version. You should upload this as a separate file labeled 'Revised Manuscript with Track Changes'.
- An unmarked version of your revised paper without tracked changes. You should upload this as a separate file labeled 'Manuscript'.

If you would like to make changes to your financial disclosure, please include your updated statement in your cover letter. Guidelines for resubmitting your figure files are available below the reviewer comments at the end of this letter.

If applicable, we recommend that you deposit your laboratory protocols in protocols.io to enhance the reproducibility of your results. Protocols.io assigns your protocol its own identifier (DOI) so that it can be cited independently in the future. For instructions see: https://journals.plos.org/plosone/s/submission-guidelines#loc-laboratory-protocols . Additionally, PLOS ONE offers an option for publishing peer-reviewed Lab Protocol articles, which describe protocols hosted on protocols.io. Read more information on sharing protocols at https://plos.org/protocols?utm_medium=editorial-email&utm_source=authorletters&utm_campaign=protocols .

We look forward to receiving your revised manuscript.

Kind regards,

Ran Barkai

Academic Editor

PLOS ONE

Journal Requirements:

When submitting your revision, we need you to address these additional requirements.

1. Please ensure that your manuscript meets PLOS ONE's style requirements, including those for file naming. The PLOS ONE style templates can be found at

https://journals.plos.org/plosone/s/file?id=wjVg/PLOSOne_formatting_sample_main_body.pdf and

https://journals.plos.org/plosone/s/file?id=ba62/PLOSOne_formatting_sample_title_authors_affiliations.pdf

2. We note that your Data Availability Statement is currently as follows: [All relevant data are within the manuscript and its Supporting Information files.]

Please confirm at this time whether or not your submission contains all raw data required to replicate the results of your study. Authors must share the “minimal data set” for their submission. PLOS defines the minimal data set to consist of the data required to replicate all study findings reported in the article, as well as related metadata and methods (https://journals.plos.org/plosone/s/data-availability#loc-minimal-data-set-definition).

For example, authors should submit the following data:

- The values behind the means, standard deviations and other measures reported;

- The values used to build graphs;

- The points extracted from images for analysis.

Authors do not need to submit their entire data set if only a portion of the data was used in the reported study.

If your submission does not contain these data, please either upload them as Supporting Information files or deposit them to a stable, public repository and provide us with the relevant URLs, DOIs, or accession numbers. For a list of recommended repositories, please see https://journals.plos.org/plosone/s/recommended-repositories.

If there are ethical or legal restrictions on sharing a de-identified data set, please explain them in detail (e.g., data contain potentially sensitive information, data are owned by a third-party organization, etc.) and who has imposed them (e.g., an ethics committee). Please also provide contact information for a data access committee, ethics committee, or other institutional body to which data requests may be sent. If data are owned by a third party, please indicate how others may request data access.

3. We note that Figure 1 in your submission contain [map/satellite] images which may be copyrighted. All PLOS content is published under the Creative Commons Attribution License (CC BY 4.0), which means that the manuscript, images, and Supporting Information files will be freely available online, and any third party is permitted to access, download, copy, distribute, and use these materials in any way, even commercially, with proper attribution. For these reasons, we cannot publish previously copyrighted maps or satellite images created using proprietary data, such as Google software (Google Maps, Street View, and Earth). For more information, see our copyright guidelines: http://journals.plos.org/plosone/s/licenses-and-copyright.

We require you to either (1) present written permission from the copyright holder to publish these figures specifically under the CC BY 4.0 license, or (2) remove the figures from your submission:

1. You may seek permission from the original copyright holder of Figure 1 to publish the content specifically under the CC BY 4.0 license. 

We recommend that you contact the original copyright holder with the Content Permission Form (http://journals.plos.org/plosone/s/file?id=7c09/content-permission-form.pdf) and the following text:

“I request permission for the open-access journal PLOS ONE to publish XXX under the Creative Commons Attribution License (CCAL) CC BY 4.0 (http://creativecommons.org/licenses/by/4.0/). Please be aware that this license allows unrestricted use and distribution, even commercially, by third parties. Please reply and provide explicit written permission to publish XXX under a CC BY license and complete the attached form.”

Please upload the completed Content Permission Form or other proof of granted permissions as an ""Other"" file with your submission.

In the figure caption of the copyrighted figure, please include the following text: “Reprinted from [ref] under a CC BY license, with permission from [name of publisher], original copyright [original copyright year].”

2. If you are unable to obtain permission from the original copyright holder to publish these figures under the CC BY 4.0 license or if the copyright holder’s requirements are incompatible with the CC BY 4.0 license, please either i) remove the figure or ii) supply a replacement figure that complies with the CC BY 4.0 license. Please check copyright information on all replacement figures and update the figure caption with source information. If applicable, please specify in the figure caption text when a figure is similar but not identical to the original image and is therefore for illustrative purposes only.

The following resources for replacing copyrighted map figures may be helpful:

USGS National Map Viewer (public domain): http://viewer.nationalmap.gov/viewer/

The Gateway to Astronaut Photography of Earth (public domain): http://eol.jsc.nasa.gov/sseop/clickmap/

Maps at the CIA (public domain): https://www.cia.gov/library/publications/the-world-factbook/index.html and https://www.cia.gov/library/publications/cia-maps-publications/index.html

NASA Earth Observatory (public domain): http://earthobservatory.nasa.gov/

Landsat: http://landsat.visibleearth.nasa.gov/

USGS EROS (Earth Resources Observatory and Science (EROS) Center) (public domain): http://eros.usgs.gov/#

Natural Earth (public domain): http://www.naturalearthdata.com/

4. If the reviewer comments include a recommendation to cite specific previously published works, please review and evaluate these publications to determine whether they are relevant and should be cited. There is no requirement to cite these works unless the editor has indicated otherwise. 

[Note: HTML markup is below. Please do not edit.]

Reviewers' comments:

Reviewer's Responses to Questions

**Comments to the Author**

1. Is the manuscript technically sound, and do the data support the conclusions?

Reviewer #1: Yes

Reviewer #2: Partly

**********

2. Has the statistical analysis been performed appropriately and rigorously?

Reviewer #1: I Don't Know

Reviewer #2: Yes

**********

3. Have the authors made all data underlying the findings in their manuscript fully available?

The PLOS Data policy

Reviewer #1: Yes

Reviewer #2: No

**********

4. Is the manuscript presented in an intelligible fashion and written in standard English?

Reviewer #1: Yes

Reviewer #2: Yes

**********

Reviewer #1: This is a very significant article, and with a few additions and some clearer wording, should definitely be published

The paper is about two related, but distinct, hypothesized phenomena. The first is that there exist “links between hunter-gatherer economic organization and emotional wellbeing”. The second is that these links may be passed on to subsequent generations that have transitioned to non-hunter-gatherer economies “through transgenerational cultural transmission”. From the author’s conclusion, it is clear that the second of these phenomena is what the paper focusses on and hopes to demonstrate. This is confirmed in the first sentence in the methodology section, which states: “This study examines how the variance in measured average population-level happiness varies with the fraction of ancestry from hunter-predominant, gather-predominant, or agricultural-predominant societies.”

However, the author needs to signal that these are two rather different phenomena, particularly in terms of what might account for them. In the case of the first hypothesis, the author briefly suggests possible explanations: that “forms of economic organization more closely tied to rhythms of natural environmental cycles contained advantages for emotional wellbeing”, and also that emotional wellbeing is related to the relative lack of inequality and “a lack of class stratification” among hunter-gatherers.

Where the author refers to the relationship between happiness and low levels of inequality, some reference is needed to the distinction between material inequality and inequality based on the existence of a status hierarchy, as these may not coincide.

In the case of the second hypothesis, the author leaves several issues only briefly examined, and in particular when. why and how the cultural transmission of the ancestral benefits for hunter-gatherer emotional well-being persist or do not persist in descendant generations. Much depends on the author’s reliance on a single 2018 source, Giuliano P, Nunn N. Ancestral Characteristics of Modern Populations. Economic History of Developing Regions. Part of the issue here is in the method to distinguish between a population with an ancestry from hunter-gatherer societies, and those without such an ancestry. This distinction is made on the basis of language, also implying that this is the mode of cultural transmission. According to Google Scholar, the Giuliano and Nunn article has been cited no less than 117 times. Given its key place in the author’s argument, I would have expected some discussion of any issues raised that are relevant to his hypothesis in this subsequent literature.

In addition, I found that in some places the wording was either a bit awkward, ungrammatical, or difficult to understand. As examples, I note the following:

“and appears to have intellectual the culture of Anglo-American analytic philosophy, including the notion of preference optimization dating to Jeremy Bentham.”

“Social theorists including Freud, Durkheim and Rousseau identified potential links between hunter-gatherer economic organization and emotional wellbeing that would not be well captured by a utility from consumption framework.”

“caution should be used in applying a causal.”

Reviewer #2: This article combines a previously-published method linking the geographical distribution of ethnic ancestry, via language groups and pre-industrial ethnographic records, with the World Values Survey life satisfaction scores. The main finding is a statistically-significant, positive relationship between the estimated fraction of Gatherer ancestry and life satisfaction, at the national level. I think this is an interesting and important finding, worthy of publication. However I think the article is not sufficiently developed at present, and there are a number of aspects that need to be strengthened so that the analyses are performed to a high technical standard and the conclusions adequately supported.

1. I think it’s important to expand the discussion of the ethnologue approach. I know the linking to the ethnography is not the new contribution of this work, but I was surprised that the original references for the method did not seem to address this aspect. Is the United States linked, in this analysis, to an English heritage because that’s the predominant language for the country? If so, how does this affect countries that strongly enforced national languages in the 19th century, like France? A bit of discussion about this seems pertinent - there might be systematic underestimates of ancestry of groups that were coerced to speak a dominant language rather than maintaining their traditional language. I think it’s quite important for the conclusions and needs to be discussed, particularly given that prior research has shown that immigrants tend to shift their life satisfaction toward their new country, even within a given lifetime (e.g. Helliwell et al., International Migration and World Happiness, World Happiness Report 2018).

2. It’s not clear to me that the binary distinctions of ‘gathering predominant’ vs. ‘hunting predominant’ is the best way to analyze the atlas data. Forager lifeways were diverse, and usually involved a combination of hunting, fishing and gathering in variable proportions (see e.g. Kelly, 2013, Lifeways of Hunter Gatherers). It would therefore be surprising that the hunting-predominant societies were not also doing a lot of gathering (and most were probably fishing too). I see that the Atlas includes a column that actually assesses all forms of subsistence - could this be used instead of the simple binary distinctions?

3. I feel the paper needs some more substantive thought into exactly how the ancestral populations may be connected to average national life satisfaction. I presume that the fractions of modern populations that are linked to either gathering or hunting are mostly very small? (A map would be very helpful, to show the % of each ancestry by country.) If so, how would it be that these small populations manage to exert significant influence on the entire national populations? There is some literature on South American societies that might be useful for this, such as regarding the Buen Vivir idea (Villalba, Buen Vivir vs. Development: a paradigm shift in the Andes? Third World Quarterly, 2014). Related to this, it also might be helpful to discuss the fact that really this is getting at populations with recent hunting and gathering ancestry, since all humans are descended 100% from hunters and gatherers if one goes back far enough in time.

4. I think it would also be worthwhile comparing with the Gallup world poll results. There is some important nuance between how people answer the Cantril ladder vs. life satisfaction questions (Nilsson, The Cantril Ladder elicits thoughts about power and wealth, Scientific Reports 2024 https://doi.org/10.1038/s41598-024-52939-y), so I don’t think it would undermine the results here if the Gallup results were not similarly correlated with hunter-gatherer ancestry. Whatever it shows, it would provide useful additional insight.

5. There are also some additional references that may prove helpful. The works by Minarro (Miñarro et al., Happy without money: Minimally monetized societies can exhibit high subjective well-being. PLoS ONE 2021 https://doi.org/10.1371/journal.pone.0244569) and Galbraith (Galbraith et al., High life satisfaction reported among small- scale societies with low incomes, PNAS 2024 https://doi.org/10.1073/pnas.2311703121) seem particularly relevant, and would tend to support the findings here with a more direct, though more locally-focused method.

6. The discussion states that ‘findings are also notable the limited quantitative contribution of hunting and gathering predominant societies, accounting for 180 of 1265 societies in the Ethnographic Atlas.’ So what were the other 1085 societies doing for subsistence?

7. The figure does not seem appropriate. This figure simply shows the previously reported WVS life satisfaction score by country, without adding anything related to the work at present. I think it would be far more useful to include a figure of the hunter and gatherer ancestry fractions by country, or similar. Perhaps the three could be shown as adjacent panels - each of the two ancestries, and the life satisfaction?

Again I think this is potentially very valuable work and hope that these comments can help to strengthen it.

**********

what does this mean? ). If published, this will include your full peer review and any attached files.

If you choose “no”, your identity will remain anonymous but your review may still be made public.

**Do you want your identity to be public for this peer review?** For information about this choice, including consent withdrawal, please see our Privacy Policy

Reviewer #1: **Yes: ** Adrian Tanner

Reviewer #2: No

**********

[NOTE: If reviewer comments were submitted as an attachment file, they will be attached to this email and accessible via the submission site. Please log into your account, locate the manuscript record, and check for the action link "View Attachments". If this link does not appear, there are no attachment files.]

While revising your submission, please upload your figure files to the Preflight Analysis and Conversion Engine (PACE) digital diagnostic tool, https://pacev2.apexcovantage.com/ . PACE helps ensure that figures meet PLOS requirements. To use PACE, you must first register as a user. Registration is free. Then, login and navigate to the UPLOAD tab, where you will find detailed instructions on how to use the tool. If you encounter any issues or have any questions when using PACE, please email PLOS at figures@plos.org . Please note that Supporting Information files do not need this step.

# Author comment: Author response to Decision Letter 1

**DOI:** 10.1371/journal.pone.0336161.r002

19 Oct 2025

This text has been uploaded seperately as part of the rebusmission, with color-coding to indicate query responses.

1. Please ensure that your manuscript meets PLOS ONE's style requirements, including those for file naming. The PLOS ONE style templates can be found at

https://journals.plos.org/plosone/s/file?id=wjVg/PLOSOne_formatting_sample_main_body.pdf and

https://journals.plos.org/plosone/s/file?id=ba62/PLOSOne_formatting_sample_title_authors_affiliations.pdf

I have reviewed the style requirements, including file naming, and to my understanding this draft is consistent with the requirements. I am very happy to make any additional adjustments if indicated.

2. We note that your Data Availability Statement is currently as follows: [All relevant data are within the manuscript and its Supporting Information files.]

Please confirm at this time whether or not your submission contains all raw data required to replicate the results of your study. Authors must share the “minimal data set” for their submission. PLOS defines the minimal data set to consist of the data required to replicate all study findings reported in the article, as well as related metadata and methods (https://journals.plos.org/plosone/s/data-availability#loc-minimal-data-set-definition).

For example, authors should submit the following data:

- The values behind the means, standard deviations and other measures reported;

- The values used to build graphs;

- The points extracted from images for analysis.

Authors do not need to submit their entire data set if only a portion of the data was used in the reported study.

All the data used in this study is publicaly available, from the four following data sources: the World Bank World Development Indicators, the World Values Survey, the supporting files of Giuliano and Nunn 2018 and supporting files of Enke 2019.

However, I have now also taken the additional step of posting the merged dataset and STATA code for production of all tables in the analyses in github: https://github.com/mfbasilico/GatheringHappiness. The posted code reproduces all tables.

If your submission does not contain these data, please either upload them as Supporting Information files or deposit them to a stable, public repository and provide us with the relevant URLs, DOIs, or accession numbers. For a list of recommended repositories, please see https://journals.plos.org/plosone/s/recommended-repositories.

If there are ethical or legal restrictions on sharing a de-identified data set, please explain them in detail (e.g., data contain potentially sensitive information, data are owned by a third-party organization, etc.) and who has imposed them (e.g., an ethics committee). Please also provide contact information for a data access committee, ethics committee, or other institutional body to which data requests may be sent. If data are owned by a third party, please indicate how others may request data access.

3. We note that Figure 1 in your submission contain [map/satellite] images which may be copyrighted. All PLOS content is published under the Creative Commons Attribution License (CC BY 4.0), which means that the manuscript, images, and Supporting Information files will be freely available online, and any third party is permitted to access, download, copy, distribute, and use these materials in any way, even commercially, with proper attribution. For these reasons, we cannot publish previously copyrighted maps or satellite images created using proprietary data, such as Google software (Google Maps, Street View, and Earth). For more information, see our copyright guidelines: http://journals.plos.org/plosone/s/licenses-and-copyright.

We require you to either (1) present written permission from the copyright holder to publish these figures specifically under the CC BY 4.0 license, or (2) remove the figures from your submission:

1. You may seek permission from the original copyright holder of Figure 1 to publish the content specifically under the CC BY 4.0 license.

We recommend that you contact the original copyright holder with the Content Permission Form (http://journals.plos.org/plosone/s/file?id=7c09/content-permission-form.pdf) and the following text:

“I request permission for the open-access journal PLOS ONE to publish XXX under the Creative Commons Attribution License (CCAL) CC BY 4.0 (http://creativecommons.org/licenses/by/4.0/). Please be aware that this license allows unrestricted use and distribution, even commercially, by third parties. Please reply and provide explicit written permission to publish XXX under a CC BY license and complete the attached form.”

Please upload the completed Content Permission Form or other proof of granted permissions as an ""Other"" file with your submission.

In the figure caption of the copyrighted figure, please include the following text: “Reprinted from [ref] under a CC BY license, with permission from [name of publisher], original copyright [original copyright year].”

2. If you are unable to obtain permission from the original copyright holder to publish these figures under the CC BY 4.0 license or if the copyright holder’s requirements are incompatible with the CC BY 4.0 license, please either i) remove the figure or ii) supply a replacement figure that complies with the CC BY 4.0 license. Please check copyright information on all replacement figures and update the figure caption with source information. If applicable, please specify in the figure caption text when a figure is similar but not identical to the original image and is therefore for illustrative purposes only.

The following resources for replacing copyrighted map figures may be helpful:

USGS National Map Viewer (public domain): http://viewer.nationalmap.gov/viewer/

The Gateway to Astronaut Photography of Earth (public domain): http://eol.jsc.nasa.gov/sseop/clickmap/

Maps at the CIA (public domain): https://www.cia.gov/library/publications/the-world-factbook/index.html and https://www.cia.gov/library/publications/cia-maps-publications/index.html

NASA Earth Observatory (public domain): http://earthobservatory.nasa.gov/

Landsat: http://landsat.visibleearth.nasa.gov/

USGS EROS (Earth Resources Observatory and Science (EROS) Center) (public domain): http://eros.usgs.gov/#

Natural Earth (public domain): http://www.naturalearthdata.com/

Thank you very much for this guidance. After reflection including Reviewer 2 Comment #7 as well as copyright limitations, I am in agreement that the figure does not add sufficiently to the analysis, and that these results are available elsewhere. I have included an additional table in the manuscript from the helpful suggestion regarding Life Satisfaction, and instead of a map I have included summary statistics within the text on the prevalence and variance of hunter and gatherer ancestry using the Ethnologue approach as suggested by this comment.

4. If the reviewer comments include a recommendation to cite specific previously published works, please review and evaluate these publications to determine whether they are relevant and should be cited. There is no requirement to cite these works unless the editor has indicated otherwise.

Thank you very much for this comment. The updated manuscript includes an expanded literature review which touches on additional themes mentioned in the reviewers and editors, including wellbeing in small-scale societies, limitations to the Ethnologue approach, and the citations suggested by Reviewer 2 in Comment #5.

[Note: HTML markup is below. Please do not edit.]

Reviewers' comments:

Reviewer's Responses to Questions

Comments to the Author

1. Is the manuscript technically sound, and do the data support the conclusions?

The manuscript must describe a technically sound piece of scientific research with data that supports the conclusions. Experiments must have been conducted rigorously, with appropriate controls, replication, and sample sizes. The conclusions must be drawn appropriately based on the data presented.

Reviewer #1: Yes

Reviewer #2: Partly

________________________________________

2. Has the statistical analysis been performed appropriately and rigorously?

Reviewer #1: I Don't Know

Reviewer #2: Yes

As mentioned above, I have included data and STATA code used for each of the tables publicly on github. Of note, the standard errors in columns 4 & 5 of Tables 1-3 have been updated to heteroskedasticity-robust as originally indicated in the methods section.

________________________________________

3. Have the authors made all data underlying the findings in their manuscript fully available?

The PLOS Data policy requires authors to make all data underlying the findings described in their manuscript fully available without restriction, with rare exception (please refer to the Data Availability Statement in the manuscript PDF file). The data should be provided as part of the manuscript or its supporting information, or deposited to a public repository. For example, in addition to summary statistics, the data points behind means, medians and variance measures should be available. If there are restrictions on publicly sharing data—e.g. participant privacy or use of data from a third party—those must be specified.

Reviewer #1: Yes

Reviewer #2: No

As mentioned above, all data used in the analysis is available from 4 publicly available datasources (World Bank WDI, World Values Survey, Enke 2019 and Giuliano-Nunn 2018). I have taken the additional step of posting merged dataset with all data elements used in the analysis, and code for the tables.

________________________________________

4. Is the manuscript presented in an intelligible fashion and written in standard English?

PLOS ONE does not copyedit accepted manuscripts, so the language in submitted articles must be clear, correct, and unambiguous. Any typographical or grammatical errors should be corrected at revision, so please note any specific errors here.

Reviewer #1: Yes

Reviewer #2: Yes

________________________________________

5. Review Comments to the Author

Please use the space provided to explain your answers to the questions above. You may also include additional comments for the author, including concerns about dual publication, research ethics, or publication ethics. (Please upload your review as an attachment if it exceeds 20,000 characters)

Reviewer #1: This is a very significant article, and with a few additions and some clearer wording, should definitely be published

The paper is about two related, but distinct, hypothesized phenomena. The first is that there exist “links between hunter-gatherer economic organization and emotional wellbeing”. The second is that these links may be passed on to subsequent generations that have transitioned to non-hunter-gatherer economies “through transgenerational cultural transmission”. From the author’s conclusion, it is clear that the second of these phenomena is what the paper focusses on and hopes to demonstrate. This is confirmed in the first sentence in the methodology section, which states: “This study examines how the variance in measured average population-level happiness varies with the fraction of ancestry from hunter-predominant, gather-predominant, or agricultural-predominant societies.”

However, the author needs to signal that these are two rather different phenomena, particularly in terms of what might account for them. In the case of the first hypothesis, the author briefly suggests possible explanations: that “forms of economic organization more closely tied to rhythms of natural environmental cycles contained advantages for emotional wellbeing”, and also that emotional wellbeing is related to the relative lack of inequality and “a lack of class stratification” among hunter-gatherers.

Where the author refers to the relationship between happiness and low levels of inequality, some reference is needed to the distinction between material inequality and inequality based on the existence of a status hierarchy, as these may not coincide.

Thank you very much for this thoughtful comment. I have provided additional discussion on the distinction between material inequality and status hierarchy, and how each could be relevant to the results of the analysis.

In the case of the second hypothesis, the author leaves several issues only briefly examined, and in particular when. why and how the cultural transmission of the ancestral benefits for hunter-gatherer emotional well-being persist or do not persist in descendant generations. Much depends on the author’s reliance on a single 2018 source, Giuliano P, Nunn N. Ancestral Characteristics of Modern Populations. Economic History of Developing Regions. Part of the issue here is in the method to distinguish between a population with an ancestry from hunter-gatherer societies, and those without such an ancestry. This distinction is made on the basis of language, also implying that this is the mode of cultural transmission. According to Google Scholar, the Giuliano and Nunn article has been cited no less than 117 times. Given its key place in the author’s argument, I would have expected some discussion of any issues raised that are relevant to his hypothesis in this subsequent literature.

Thank you very much for this important comment. I have expanded the discussion of critiques of the Ethnologue approach within the Introduction and Discussion sections, within the section on study limitations. This approach has been used in dozens of papers within the historical economics literature; however, as you note there are important limitations, notably the assignment of a language group to an observation in the ethnographic atlas, and the use of language as the sole connection to ancestry in contemporary nation-states.

In addition, I found that in some places the wording was either a bit awkward, ungrammatical, or difficult to understand. As examples, I note the following:

“and appears to have intellectual the culture of Anglo-American analytic philosophy, including the notion of preference optimization dating to Jeremy Bentham.”

“Social theorists including Freud, Durkheim and Rousseau identified potential links between hunter-gatherer economic organization and emotional wellbeing that would not be well captured by a utility from consumption framework.”

“caution should be used in applying a causal.”

Thank you very much for these helpful examples. I edited each of areas mentioned to use clearer wording. I have also reviewed the manuscript for similar instances where the language could be clearer and adjusted accordingly.

Reviewer #2: This article combines a previously-published method linking the geographical distribution of ethnic ancestry, via language groups and pre-industrial ethnographic records, with the World Values Survey life satisfaction scores. The main finding is a statistically-significant, positive relationship between the estimated fraction of Gatherer ancestry and life satisfaction, at the national level. I think this is an interesting and important finding, worthy of publication. However I think the article is not sufficiently developed at present, and there are a number of aspects that need to be strengthened so that the analyses are performed to a high technical standard and the conclusions adequately supported.

1. I think it’s important to expand the discussion of the ethnologue approach. I know the linking to the ethnography is not the new contribution of this work, but I was surprised that the original references for the method did not seem to address this aspect. Is the United States linked, in this analysis, to an English heritage because that’s the predominant language for the country? If so, how does this affect countries that strongly enforced national languages in the 19th century, like France? A bit of discussion about this seems pertinent - there might be systematic underestimates of ancestry of groups that were coerced to speak a dominant language rather than maintaining their traditional language. I think it’s quite important for the conclusions and needs to be discussed, particularly given that prior research has shown that immigrants tend to shift their life satisfaction toward their new country, even within a giv

Attachment Submitted filename: Response to Reviewers.pdf

# Editor report: Decision Letter 1

**DOI:** 10.1371/journal.pone.0336161.r003

**Authors:** Ran Barkai

21 Oct 2025

Gatherer Ancestry Associated with National Happiness

PONE-D-25-39711R1

Dear Dr. Basilico,

We’re pleased to inform you that your manuscript has been judged scientifically suitable for publication and will be formally accepted for publication once it meets all outstanding technical requirements.

Within one week, you’ll receive an e-mail detailing the required amendments. When these have been addressed, you’ll receive a formal acceptance letter and your manuscript will be scheduled for publication.

An invoice will be generated when your article is formally accepted. Please note, if your institution has a publishing partnership with PLOS and your article meets the relevant criteria, all or part of your publication costs will be covered. Please make sure your user information is up-to-date by logging into Editorial Manager at Editorial Manager®  and clicking the ‘Update My Information' link at the top of the page. For questions related to billing, please contact billing support .

If your institution or institutions have a press office, please notify them about your upcoming paper to help maximize its impact. If they’ll be preparing press materials, please inform our press team as soon as possible -- no later than 48 hours after receiving the formal acceptance. Your manuscript will remain under strict press embargo until 2 pm Eastern Time on the date of publication. For more information, please contact onepress@plos.org.

Kind regards,

Ran Barkai

Academic Editor

PLOS ONE

Additional Editor Comments (optional):

Reviewers' comments:

# Editor report: Acceptance letter

**DOI:** 10.1371/journal.pone.0336161.r004

**Authors:** Ran Barkai

PONE-D-25-39711R1

PLOS ONE

Dear Dr. Basilico,

I'm pleased to inform you that your manuscript has been deemed suitable for publication in PLOS ONE. Congratulations! Your manuscript is now being handed over to our production team.

At this stage, our production department will prepare your paper for publication. This includes ensuring the following:

* All references, tables, and figures are properly cited

* All relevant supporting information is included in the manuscript submission,

* There are no issues that prevent the paper from being properly typeset

You will receive further instructions from the production team, including instructions on how to review your proof when it is ready. Please keep in mind that we are working through a large volume of accepted articles, so please give us a few days to review your paper and let you know the next and final steps.

Lastly, if your institution or institutions have a press office, please let them know about your upcoming paper now to help maximize its impact. If they'll be preparing press materials, please inform our press team within the next 48 hours. Your manuscript will remain under strict press embargo until 2 pm Eastern Time on the date of publication. For more information, please contact onepress@plos.org.

You will receive an invoice from PLOS for your publication fee after your manuscript has reached the completed accept phase. If you receive an email requesting payment before acceptance or for any other service, this may be a phishing scheme. Learn how to identify phishing emails and protect your accounts at https://explore.plos.org/phishing.

If we can help with anything else, please email us at customercare@plos.org.

Thank you for submitting your work to PLOS ONE and supporting open access.

Kind regards,

PLOS ONE Editorial Office Staff

on behalf of

Professor Ran Barkai

Academic Editor

PLOS ONE
